# Supplementary material for: Cumulative cgMLST provides increased discrimination of nested phylogenetic groups
Source: Genome Med. 2026 Jul 24;18:157. doi: 10.1186/s13073-026-01720-z (PMC13682010; doi:10.1186/s13073-026-01720-z)
Supplement: Supplementary file 1 [file 13073_2026_1720_MOESM1_ESM.docx]

**Supplementary material**

**Cumulative cgMLST schemes for increased discrimination of nested phylogenetic groups**

**Armen Ovsepian^1^, Jose F. Delgado-Blas^1^, Martin Rethoret-Pasty^1^, Melissa J. Martin^2^, François Lebreton^2^, and Sylvain Brisse^1^ ***

^1^ Institut Pasteur, Université Paris Cité, Biodiversity and Epidemiology of Bacterial Pathogens, Paris, France

^2^ Multidrug-Resistant Organism Repository and Surveillance Network (MRSN), Diagnostics and Countermeasures Branch, CIDR, Walter Reed Army Institute of Research, Silver Spring, Maryland, USA

*Correspondence:

Sylvain Brisse: Institut Pasteur, Biodiversity and Epidemiology of Bacterial Pathogens, 25-28 rue du Docteur Roux, F-75724, Paris, France; Phone: +33 1 45 68 83 34; E-mail: [sylvain.brisse@pasteur.fr](mailto:sylvain.brisse@pasteur.fr)

Contents

[Fig. S1	3](#_Toc234077342)

[Table S1	4](#_Toc234077343)

[Fig. S2	5](#_Toc234077344)

[Fig. S3	6](#_Toc234077345)

[Fig. S4	7](#_Toc234077346)

[Fig. S5	8](#_Toc234077347)

[Supplementary Text S1. Description of the SL147 cgMLST scheme	8](#_Toc234077348)

[Fig. S6	9](#_Toc234077349)

[Fig. S7	10](#_Toc234077350)

[Fig. S8	11](#_Toc234077350)

[Fig. S9	12](#_Toc234077351)

[Supplementary Text S2. Description of the SL307 cgMLST scheme	13](#_Toc234077352)

[Fig. S10	14](#_Toc234077353)

[Fig. S12	16](#_Toc234077354)

[Fig. S13	17](#_Toc234077355)

[Fig. S14.	18](#_Toc234077356)

Fig. S1. Number of missing alleles out of the 2,752 loci of the Kpn-cgMLST scheme for the BIGSdb-Pasteur isolates SB5514 (A), SB5734 (B), SB5806 (C) at 30X to 90X genome coverage, in triplicates.

Table S1: The number of isolates collected from each country by the Multidrug-Resistant Organism Repository and Surveillance Network from 2003 until 2021 (dataset 8).

| **Country** | **Number of samples** |
| --- | --- |
| Thailand | 363 |
| Georgia | 359 |
| Peru | 305 |
| Jordan | 218 |
| Kenya | 159 |
| USA | 97 |
| Uganda | 36 |
| Philippines | 25 |
| Ukraine | 7 |
| Germany | 5 |
| Turkey | 4 |
| Italy | 3 |
| Israel | 2 |
| South Korea | 2 |
| Afghanistan | 1 |
| Guam | 1 |

Fig. S2. Schematic presentation of the main steps followed for the development of the core genome MLST scheme for *Klebsiella pneumoniae sensu stricto* (Kpn-cgMLST).

Fig. S3. 2D-plot showing the relationships between the locus length (bp) and the number of alleles, for all the Kpn-cgMLST loci (n=2752). As locus length, the length of the type allele was used. At the central panel, each point represents a locus; the x axis corresponds to the locus length and the y axis to the number of alleles of that locus. At top/bottom and right/left panels the distributions (100 bins) and the boxplots of each parameter are shown, respectively.

Fig. S4**.** The allele call rate (expressed as %) of the Kpn-cgMLST scheme loci (n=2752) in the tested datasets. (A) the 2,483 setup isolates for the creation of the scheme; (B) the 1,969 isolates from the EuSCAPE project; (C) the 1,705 *K. pneumoniae* isolates from the SpARK project; (D) the 1,587 *K. pneumoniae* isolates collected from 2003 until 2021 by the Multidrug-Resistant Organism Repository and Surveillance Network; (E) the 2,520 *K. pneumoniae* isolates collected from multiple ecological sources from 2001 until 2020 in a One Health study in Norway. Bin size=0.1. Note that the X-axis starts at 90%.

Fig. S5. Minimum spanning tree based on cgMLST distances between the n=37 isolates described in dataset 9 in **Methods**. The distances were calculated based on the Kpn scheme loci (n=2752), plus the KpSC scheme (n=629) and the rMLST (n=53) loci. The colors of the nodes (isolates) indicate the cluster of the isolates; isolates connected with distances < 15 belong to the same cluster as defined in (34). Singletons were not considered clusters.

# Supplementary Text S1. Description of the SL147 cgMLST scheme

In the setup dataset of 1,069 genomes, locus lengths ranged from 90 to 4,053 bp, with a total of 1 to 50 alleles per locus, and allele number increasing with locus size (**Additional file 1: Fig. S6**). The locus names, type allele sequences, locus lengths, allowed length ranges, gene names, and gene products are provided in **Additional file 4**. The mean allele call rate per isolate for the setup isolates was 99.1 ± 1.2 %, with 24 isolates (2.2 %) having ≤ 95% allele call rate (**Additional file 1: Fig. S7A**). The mean allele call rate for the 1,404 isolates of the validation dataset was 99.0 ± 1.6 %, with 42 isolates (3.0 %) having ≤ 95% allele call rate (**Additional file 1: Fig. S7B**).

Fig. S6. 2D-plot showing the relationships between the locus length (bp) and the number of alleles, for all the SL147-cgMLST loci (n=852). As locus length, the length of the type allele was used. At the central panel, each point represents a locus; the x axis corresponds to the locus length and the y axis to the number of alleles of that locus. At top/bottom and right/left panels the distributions (100 bins) and the boxplots of each parameter are shown, respectively.

 Fig. S7. The allele call rate (expressed as %) of the SL147 cgMLST scheme loci (n=852) obtained using (A) the 1,069 setup isolates (used for the creation of the SL147 cgMLST scheme); or (B) the 1,404 isolates of the validation dataset. Bin size=0.1. The X-axis starts at 80% for both panels.

Fig. S8. The effect of the inclusion of the SL147-specific loci on the pairwise allelic distances between the validation dataset isolates (n=1404). The distances were calculated based on the KpSC (n=629), the MLST (n=7), and the rMLST (n=53) scheme loci together (labeled ‘KpSC’ on the figure for simplicity), or with the addition of the Kpn (n=2752) and the SL147 (n=852) scheme loci, in which cases the 7 MLST loci were excluded. (**A**) The distribution of pairwise distances between the isolates by using only the KpSC (blue), or additionally the Kpn (red) and then additionally the SL147 (green) cgMLST loci. (**B**, **C**, **D**): Violin plots of the intra- (blue) and inter-ST (red) pairwise distances by using only the KpSC scheme (**B**), or additionally the Kpn scheme (**C**) and then additionally the SL147 scheme (**D**). The nonparametric Mann-Whitney U test was used to determine whether there was a significant difference between the distributions of the intra- and inter- group pairwise allelic distances (*=p≤0.05, **=p≤0.01, and ***=p≤0.001). (**E**) Relationships between pairwise distances derived with or without the inclusion of the Kpn scheme; (**F**) Comparison of pairwise distances with or without the inclusion of the SL147 scheme loci. In E and F, the dashed grey line corresponds to y=x, and the red line corresponds to the linear regression by applying the least squares method.

Fig. S9. Minimum spanning trees based on cgMLST distances between the n=114 SL147 outbreak isolates described in **Methods** (dataset 12). The distances were calculated based on the KpSC scheme (n=629) (plus the MLST (n=7) and the rMLST (n=53) loci) (**A**) or with the addition of the Kpn scheme loci (n=2752) (**B**), or also additionally with the SL147 scheme loci (**C**), in which cases the 7 MLST loci were excluded. The colors applied to the trees correspond to single linkage clusters defined using the whole genome SNP distances, with a cut-off value of 4 SNPs.

# Supplementary Text S2. Description of the SL307 cgMLST scheme

In the reference dataset of 1,080 genomes, locus lengths ranged from 90 to 4,278 bp, with one to 33 alleles per locus, and the number of alleles increased with locus size (**Additional file 1: Fig. S10**). **Additional file 5** provides detailed information on locus names, type allele sequences, locus lengths, allowed length ranges, gene names, and gene products. The mean allele call rate per isolate for the SL307-cgMLST setup isolates was 99.4 ± 1.0 %, with 8 isolates (0.7 %) having ≤ 95% allele call rate (**Additional file 1: Fig. S11A**). The mean allele call rate for the 947 isolates of the validation dataset was 99.3 ± 1.4 %, with 19 isolates (1.9 %) having ≤ 95% allele call rate (**Additional file 1: Fig. S11B**).

Fig. S10. 2D-plot showing the relationships between the locus length (bp) and the number of alleles, for all the SL307-cgMLST loci (n=947). As locus length, the length of the type allele was used. At the central panel, each point represents a locus; the x axis corresponds to the locus length and the y axis to the number of alleles of that locus. At top/bottom and right/left panels the distributions (100 bins) and the boxplots of each parameter are shown, respectively.

Fig. S11. The allele-call rate of the SL307-cgMLST scheme (n=947) in the tested dataset. (A) the 1,080 setup isolates for the creation of the SL307 core genome MLST scheme; (B) the 947 isolates of the validation dataset. Bin size=0.1. The X-axis starts at 80% for both panels.

Fig. S12. The effect of the inclusion of the SL307-specific loci on the pairwise allelic distances between the SL307-cgMLST validation isolates. The distances were calculated based on the KpSC (n=629), the MLST (n=7), and the rMLST (n=53) scheme loci together (labeled ‘KpSC’ on the figure for simplicity), or with the addition of the Kpn (n=2752) and the SL147 (n=947) scheme loci, in which cases the 7 MLST loci were excluded. (**A**) The distribution of pairwise distances between the isolates by using only the KpSC (blue), or additionally, the Kpn (red) and then additionally the SL307 (green) cgMLST loci. (**B**) Relationships between pairwise distances derived with or without the inclusion of the Kpn scheme; (**C**) Comparison with or without the inclusion of the SL307 scheme loci. In **B** and **C**, the dashed grey line corresponds to y=x, and the red line corresponds to the linear regression by applying the least squares method.

Fig. S13. Minimum spanning trees based on the cgMLST distances between n=48 *K. pneumoniae* SL307 outbreak isolates described in **Methods** (dataset 13). The distances were calculated based on the KpSC scheme (n=629) (plus the MLST (n=7) and the rMLST (n=53) loci) (**A**) or with the addition of the Kpn scheme loci (n=2752) (**B**), or additionally the SL307 scheme loci (n=947) (**C**), in which cases the 7 MLST loci were excluded. The colors applied to the trees correspond to single linkage clusters defined using the whole genome SNP distances, with a cut-off value of 4 SNPs.

Fig. S14. (**A**) Available schemes in BIGSdb-Pasteur for the genotyping of *K. pneumoniae* species complex (KpSC) isolates within the framework of cumulative-cgMLST strategy. (**B**) Selection of schemes in the BIGSdb-Pasteur database (56) depending on the isolates under investigation (**1**: any KpSC isolate; **2**: any Kpn isolate; **3**: Kpn SL147 isolates; **4**: Kpn SL307 isolates).
